# Supplementary material for: Toxicity Study on Crude Alkaloid Extracts of Houttuyniae herba Based on Zebrafish and Mice
Source: Molecules. 2024 Mar 1;29(5):1107. doi: 10.3390/molecules29051107 (PMC10934671; doi:10.3390/molecules29051107)
Supplement: Supplementary file 1 [file molecules-29-01107-s001.zip › molecules-2792626-supplementary.pdf]

Communication

Toxicity Study on Crude Alkaloid Extracts of *Houttuyniae herba* Based on Zebrafish and Mice

Jing Liu [1], Yingxue Wu [1], Yanni Xu [2], Ying Han [2], Shuai Kang [1], Zhong Dai [1], Hongyu Jin [1,*], Feng Wei [1,*] and Shuangcheng Ma [1]

1 National Institutes for Food and Drug Control, Beijing 100050, China; liujing_zsm@126.com (J.L.)
2 Institute of Medical Biotechnology, Chinese Academy of Medical Sciences, Beijing 100050, China
* Correspondence: jhyu@nifdc.org.cn (H.J.); weifeng@nifdc.org.cn (F.W.)

The tissue anatomy maps of the administered mice were as follows. Figure S1 and S2 were for administration of single dose of 500 mg/kg and 2000 mg/kg, respectively. While Figure S3 was for continuous administration of 2000 mg/kg, twice a day for 7 days.

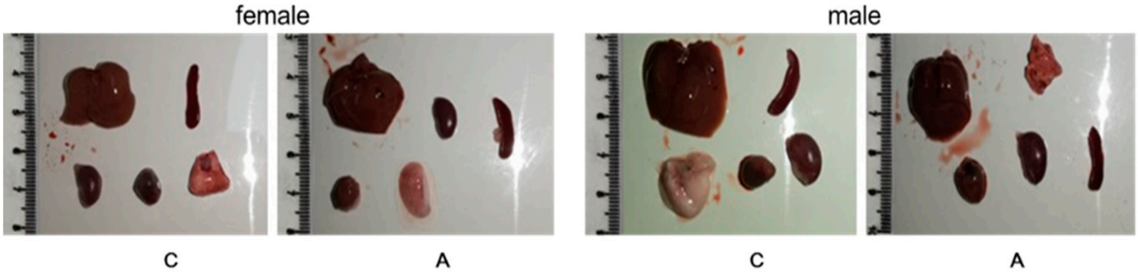

Figure S1. The tissue anatomy map.

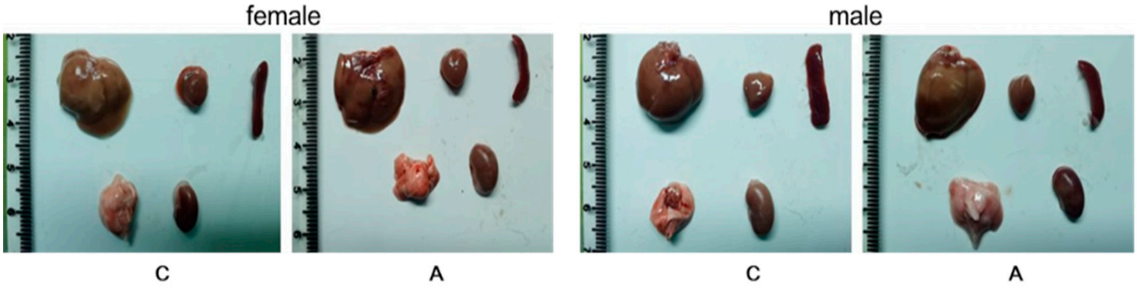

Figure S2. The tissue anatomy map.

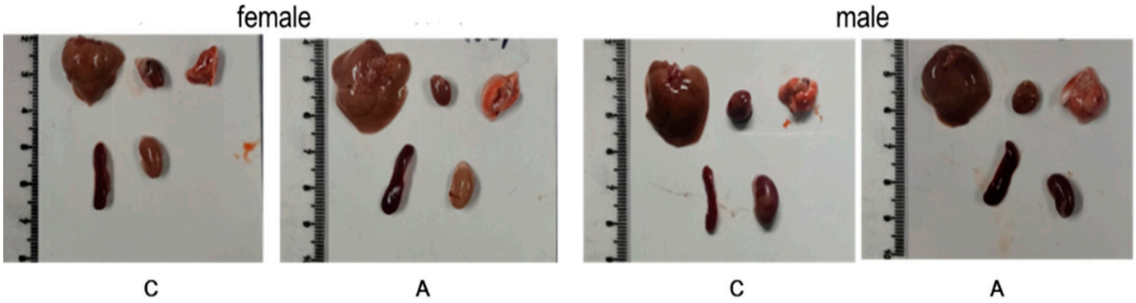

Figure S3. The tissue anatomy map.
